# Cryptococcal Osteomyelitis of the Left Acetabulum: A Case Report

**DOI:** 10.2174/1573405619666221125103107

**Published:** 2023-05-17

**Authors:** Changning Zhou, Jing Zhang, Yang Chen, Xing Ding, Fen Chen, Kai Feng, Kuntao Chen

**Affiliations:** 1 Department of Radiology, The Fifth Affiliated Hospital of Zunyi Medical University, Zhufengdadao No.1439, Doumen District, Zhuhai 519110, Guangdong, China;; 2 Second Clinical School, Zunyi Medical University, Jinwan road No.368, Jinwan District, Zhuhai 519110, Guangdong, China

**Keywords:** Cryptococcus, osteoarticular cryptococcosis, osteomyelitis, skeletal infection, acetabulum, case report

## Abstract

***Background*:** Cryptococcus, as a classical “opportunistic” fungal pathogen, is capable of disseminating an invasive infection in immunocompromised hosts. The primary sites of infection include the respiratory and central nervous systems, and skeletal infection was rarely reported. In this case, we describe a case of cryptococcal osteomyelitis involving the left side of the acetabulum in a Chinese patient with chronic hepatitis B.

***Case Presentation*:** We retrospectively reviewed the case of a female (with chronic hepatitis B) with left acetabulum pain and limited mobility, with fever occurring during the infection who presented to the Fifth Affiliated Hospital of Zunyi Medical University. Upon imaging, we found osteolytic bone destruction in the left acetabulum with inflammatory changes in the surrounding bone and soft tissue, accompanied by abscess formation. Following an 11-month of antifungal therapy, the clinical symptoms improved and the lesion area reduced in size. In addition, there was no sign of recurrence.

***Conclusion*:** Cryptococcus infections should be considered in the differential diagnosis of infectious osteolytic bone lesions, particularly when patients with immune insufficiency. Pathological examinations and fungal cultures are essential to provide a differential diagnosis.

## 1 INTRODUCTION

Cryptococcus is an opportunistic pathogenic fungus, and as such, the development of cryptococcosis is closely related to the interaction between fungal agents and host immunity. Cryptococcal infection is usually found in immunocompromised patients, especially those with cell-mediated immune deficiencies, and affects their respiratory system and central nervous system [1-3]. However, once fungal propagules are inhaled, they can affect virtually any organ through lymphangitic and hematogenous spread [4].

Skeletal cryptococcosis is rare, being found in less than 10% of patients with disseminated cryptococcosis [5], and isolated skeletal cryptococcal infections are found in an even lower percentage of patients. Although any bone or joint can be affected, the most common site of skeletal cryptococcosis is the vertebrae, the sufficient blood supply of the vertebrae might explain this finding. The next most common sites are the skull and femur, and the most commonly affected joint is the knee [4]. Isolated cryptococcal infections that occur in the acetabulum are extremely rare, and as far as we know, so far there has been no case report of cryptococcosis involving only the acetabulum. Here, we present a case of cryptococcosis involving the left side of the acetabulum in order to improve the clinical and imaging understanding of the disease.

## 2 CASE PRESENTATION

In this case, a 31-year-old female presented with progressive pain and decreased range of motion of her left hip over 2 months, and the pain had increased progressively and worsened with activity. During the 2 months, the patient reported fever and had self-administered cephalosporin antibiotics. She had no history of chills, fatigue, weight loss, or other systemic symptoms. However, she mentioned her husband was being treated for “tuberculosis”. Physical examination revealed pain in flexion, adduction, and external rotation of the left acetabulum, but no swelling, local skin damage, ecchymosis, or obvious local deformity. It showed no respiratory and neurologic symptoms or signs. Laboratory investigations showed a serum C-reactive protein level of 67.51 mg/L, total white blood cell count of 7700/mm^3^ with 86.8% neutrophils, and erythrocyte sedimentation rate of 64 mm/h. The viral serology tests for hepatitis B yielded positive results and suggested chronic hepatitis B. The HIV screening, Mycobacterium tuberculosis antibody, and Mycobacterium tuberculosis DNA tests were all negative.

**Fig. (1):**
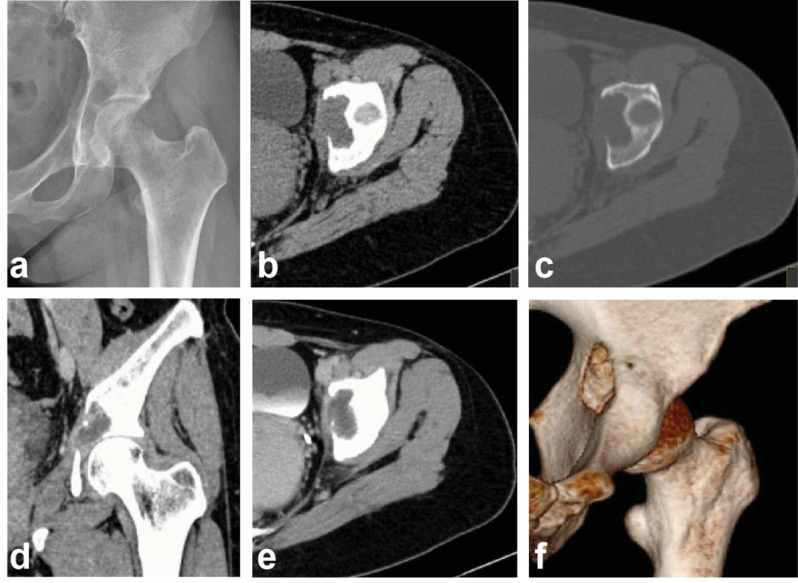
(**a**) Left hip joint radiograph. Lytic lesion was visualized in the left acetabulum, and the continuity of the local cortical bone was interrupted. Left hip joint CT: (**b**) Axial soft tissue window. (**c**) Axial bone window. (**d**) Coronal contrast-enhanced CT. (**e**) Axial contrast-enhanced CT. (**f**) Three-dimensional reconstruction. Osteolytic bone destruction and a soft tissue mass were observed in the left acetabulum. The wall was strengthened and there was a no enhancement liquid low-density shadow in the mass.

**Fig. (2):**
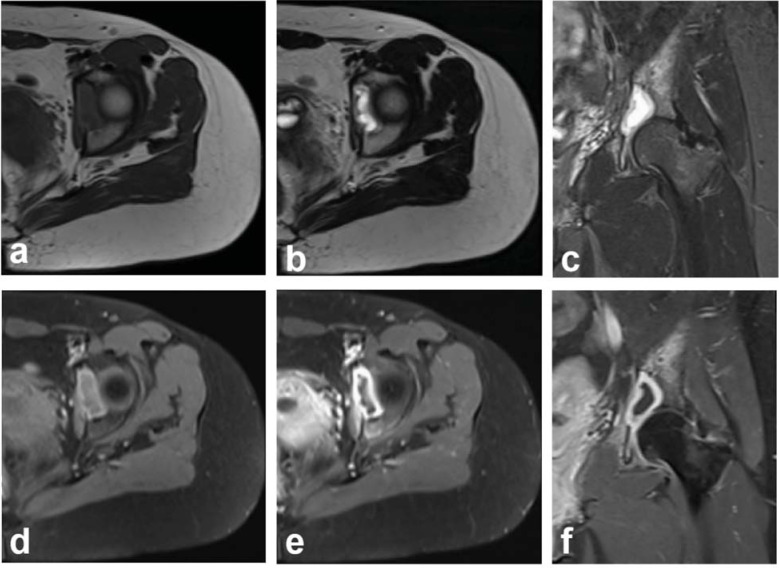
MRI of the left hip joint before treatment. (**a**) Axial image of T1-weighted image. (**b**) Axial image of T2-weighted image. (**c**) Coronal image of T2-weighted image. (**d**-**f**) Contrast-enhanced T1-weighted images. MRI showed irregular bone destruction with cortex erosion in the left acetabulum, the abscess showed a low signal intensity on the T1-weighted image and high signal intensity on the T2-weighted image,adjacent bone and soft tissue edema. The abscess wall was strengthened and the pus was not strengthened.

Radiographs demonstrated a 3 cm purely lytic lesion in the left acetabulum without sclerosis or periosteal reaction. The continuity of the local cortical bone was interrupted, and the adjacent soft tissues were inflamed (Fig. **1a**). Computed tomography (CT) scan showed osteolytic bone destruction of the left acetabulum with clear borders and no sclerosed margins, and a soft tissue mass measuring 3.5 cm by 2.0 cm by 3.3 cm in the left acetabulum. The average CT value was approximately 44HU (Fig. **1b**, **c**). The wall was strengthened with a CT value of 76HU, and there was a liquid low-density shadow in the mass with a CT value of 23HU, and no enhancement was seen (Fig. **1d**, **e**). Three-dimensional reconstruction showed bone destruction in the left acetabulum (Fig. **1f**). Chest CT showed bullae at the apical segment of the right upper lobe, with the remainder of the chest showing no obvious abnormalities. Magnetic resonance imaging (MRI) showed irregular bone destruction and abscess formation in the left acetabulum, with the abscess measuring 3.6 cm by 1.9 cm by 3.5 cm, cortex erosion and significant bone and soft tissue edema were also observed (Fig. **2a-c**). The presence of enhancement at the edge of the lesion and an area of liquefaction necrosis without enhancement in the center indicate abscess formation (Fig. **2d-f**). Imaging diagnosis of infectious disease with abscess formation.

**Fig. (3):**
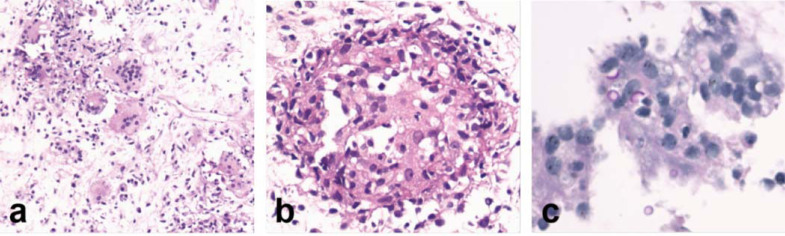
Histopathological findings of hematoxylin and eosin staining and periodic acid-Schiff’s reaction. (**a**) 200×magnification, (**b**) 400×magnification. Histopathological findings revealed granulomatous inflammation surrounded by a large amount of multinuclear giant cell, some lymphocytes, and plasma cells. (**c**) 400×magnification. Periodic acid-Schiff’s reaction can saw Cryptococcus.

The mass was sampled by fine-needle aspiration biopsy under ultrasound guidance. Histopathological examination of the specimen showed granulomatous inflammation surrounded by a large number of multinuclear giant cells, some lymphocytes, and plasma cells (Fig. **3a**, **b**), and the presence of Cryptococcus was confirmed by periodic acid-Schiff's reaction (Fig. **3c**). The resulting histopathological diagnosis was granuloma with Cryptococcus.

**Fig. (4):**
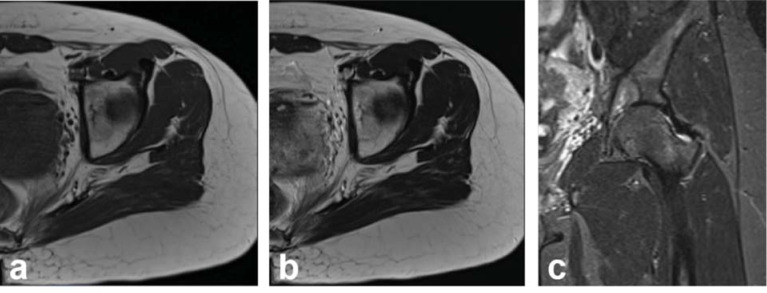
Changes in MRI images after 11 months of antifungal therapy. (**a**) Axial image of T1-weighted image. (**b**) Axial image of T2-weighted image. (**c**) Coronal image of T2-weighted image. MRI showed a reduction in the size of the lesion, but there was still some bone defect in the left acetabulum.

After one month of antifungal treatment with fluconazole and flucytosine, a fungal culture from diseased tissue secretions produced a negative result. Upon discharge, the left leg was placed under traction and immobilized to prevent hip dislocation and relieve the pain, and we continued the antifungal treatment. A follow-up of MRI after 10 months confirmed a decrease in the lesion’s size (from 3.6 cm by 1.9 cm by 3.5 cm to approximately 2.7 cm by 1.6 cm by 3.1 cm), but there was still some bone defect in the left acetabulum (Fig. **4**).

## 3 DISCUSSION

Skeletal cryptococcosis mainly manifests as chronic osteomyelitis, with systemic and local symptoms such as fever, soft tissue swelling and tenderness, and joint dysfunction [6]. Our patient presented with pain and a limited range of motion in the left hip and reported fever during the disease, which was consistent with other cases of cryptococcal osteomyelitis in the literature. The white blood cell count is often normal and the erythrocyte sedimentation rate can be elevated in patients with cryptococcal osteomyelitis [7], and we also confirmed this in this report. However, neither of these results is specific to cryptococcal skeletal infections and could also be indicative of infections in other locations or due to other organisms.

Upon imaging, cryptococcal osteomyelitis generally appears as osteolytic bone destruction, usually, without sclerosis or periosteal reaction, destruction of adjacent bone cortex and soft tissue abscesses are often seen [8, 9]. In some cases, the sclerotic margins are also imaged, as well as the sequestrum in the center of the lesion [10, 11]. Pathological fracture is sometimes visible [12, 13]. In our case, cortical bone destruction and soft tissue abscess were evident, but there were no sclerotic margins, sequestrum, and periosteal reactions. This appearance may be related to an early stage of inflammation and the duration of the disease is not long enough. Differential diagnoses that were excluded from our patient included various infectious diseases, such as other fungi, coccidiosis, blastomycosis, actinomyces and mycobacteria. When an extensive periosteal reaction occurs, it needs to be differentiated from malignant tumors. There are reports of cryptococcal osteomyelitis cases that had been initially misdiagnosed as Ewing sarcoma or osteosarcoma [14, 15].

Our patient’s history included fever and self-administered cephalosporin antibiotics during her disease. Laboratory results showed elevated C-reactive protein and erythrocyte sedimentation rate, suggesting an inflammatory reaction, and the final pathology confirmed a cryptococcal infection. Hepatic failure is considered one possible cause of immune insufficiency [16], and hepatitis B virus infection is the major cause of hepatic failure in China. Concomitant chronic hepatitis B with Cryptococcus osteomyelitis has also been reported [7]. The patient in this case had a history of chronic hepatitis B, possibly being associated with the patient's morbidity. The patient’s symptoms improved after a period of antifungal therapy and the subsequent fungal culture of secretions tested negative. Follow-up imaging at 11 months showed a reduction in the lesion’s extent and there was no sign of recurrence.

## CONCLUSION

Skeletal cryptococcosis is clinically rare, and its clinical symptoms and imaging findings lack specificity. We propose that cryptococcal osteomyelitis, along with other fungal infections, should be considered as a differential diagnosis in any patient with osteolytic lesions on imaging, Particularly, when patients with immune insufficiency present with infectious osteolytic bone lesions without periosteal reaction. The definitive diagnosis still depends on the histopathology and a fungal culture test. Through timely and accurate diagnosis and active treatment, most patients have a good prognosis.

## Data Availability

All data generated or analyzed during this study are included in this published article.

## LIST OF ABBREVIATIONS

CT: Computed Tomography
MRI: Magnetic Resonance Imaging

## STANDARDS OF REPORTING

CARE guidelines were followed.
